# Mismatch Between the Distribution of Neuromuscular Specialists and Myasthenia Gravis Patients in the US Medicare Fee‐for‐Service Population

**DOI:** 10.1002/mus.70345

**Published:** 2026-07-27

**Authors:** Lauren Herrera, David Bruckman, Ikjae Lee, Jesse D. Schold, Benjamin Claytor, Nicholas Silvestri, Yuebing Li, Michael K. Hehir

**Affiliations:** ^1^ Department of Neurology Vanderbilt University Medical Center Nashville Tennessee USA; ^2^ Center for Populations Health Research, Division of Quantitative Health Sciences Cleveland Clinic Foundation Cleveland Ohio USA; ^3^ Department of Neurology Columbia University Irving Medical Center New York New York USA; ^4^ Department of Epidemiology Colorado School of Public Health Aurora Colorad USA; ^5^ Department of Neurology Cleveland Clinic Foundation Cleveland Ohio USA; ^6^ Department of Neurology University at Buffalo Jacobs School of Medicine & Biomedical Sciences Buffalo New York USA; ^7^ Department of Neurological Sciences University of Vermont Burlington Vermont USA

**Keywords:** access to care, geographic access, myasthenia gravis, neuromuscular specialists

## Abstract

**Introduction/Aims:**

There is a mismatch between clinical need and access to neurologists across the US. Myasthenia gravis (MG) incidence and prevalence are increasing, particularly in US patients older than 65 years, raising concern for access to neuromuscular specialists that represent only 4% of neurologists. We sought to compare the prevalence of MG patients over 65 years of age to the number of board‐certified neuromuscular physicians (BCNMP) across the US.

**Methods:**

Utilizing Medicare fee‐for‐service Parts A/B coverage non‐HMO claims data, MG cases were ascertained using a validated algorithm. Number of BCNMP per state was determined using American Board of Psychiatry and Neurology and American Board of Physical Medicine and Rehabilitation resources. MG prevalence, BCNMP number, and ratios of cases per BCNMP were calculated by state, census division and region for 2012–2013 and 2018–2019. Ratios between periods were tested using Poisson regression.

**Results:**

The number of MG cases increased from 29,038 in 2012–2013 to 35,691 in 2018–2019. BCNMP increased from 585 in 2012–2013 to 806 in 2018–2019. At the national level, the ratio of Medicare MG cases per BCNMP improved from 49.5 in 2012–2013 to 44.3 in 2018–2019. The south region had highest ratios at both timeframes, while the northeast had the lowest ratios. Six states had no BCNMP at both timeframes.

**Discussion:**

While the number of BCNMP has increased nationally, supply and demand may be unequally distributed. Regions across the US demonstrate disparate ratios of MG cases per BCNMP.

**Abbreviations**
BCNMP: Board‐certified neuromuscular physician
FFS: Fee‐for‐service
MG: Myasthenia gravis
MG ADL: Myasthenia gravis activities of daily living scale
MG QOL15: Myasthenia gravis quality of life 15 scale

## 1 Introduction

Several studies demonstrate a mismatch between clinical need and access to neurologist care across the United States [1, 2]. Myasthenia gravis (MG) is a rare disorder; however, trends of growing incidence and prevalence have been noted globally and within the US, particularly in patients older than 65 years [3, 4, 5].

The goal of this study is to determine the number of board‐certified neuromuscular physicians (BCNMP) in the US caring for the population of MG patients and by extension other neuromuscular patients.

## 2 Methods

This was a retrospective, cross‐sectional, geographic analysis of the number of Medicare Fee for Service (FFS) registered MG patients compared to the number of BCNMP in the US. Medicare FFS population was chosen due to the investigators' access to Centers for Medicare and Medicaid Services (CMS) data for another study [3]. All data was collected for two timeframes, 2012–2013 and 2018–2019 to assess how mismatch may be evolving; 2018–2019 was the latest period available in the dataset. The 2‐year ranges were necessary to implement validated MG identification methodology [4]. Due to the implementation of board certification for Neuromuscular Medicine in 2008, data were analyzed after 2012 to allow for stability of data following certification initiation.

### 2.1 MG Cases

Utilizing Medicare FFS, Parts A and B coverage (FFS/AB) non‐health maintenance organization (non‐HMO) claims data, MG cases were ascertained using a validated algorithm of two MG claims within a two‐year period, from two outpatient visits or a combination of one inpatient admission and one outpatient visit, separated by at least 28 days [4, 6]. Beneficiaries with Medicare Advantage (Part C or HMO claims) were excluded as claim detail was insufficient to accurately identify MG cases.

MG prevalence was calculated by state, Census region, and Census division [7, 8].

### 2.2 Physicians

The number of BCNMP in each state was determined using verifyCERT, made available by the American Board of Psychiatry and Neurology (ABPN) or the American Board of Physical Medicine and Rehabilitation (ABPMR) Certified Physician Search. Data was sorted by state and certificate title “neuromuscular medicine.” Physicians with current, unexpired certification were included. Expired certifications were excluded due to the uncertainty of these physicians' clinical activity status. ABPN clinical neurophysiology (CNL) certified physicians were excluded due to the heterogeneous nature of this group; many of these physicians specialize in epilepsy and are not experts in MG care. American Board of Electrodiagnostic Medicine (ABEM) certified physicians were excluded as this group contains a large number of physicians who did not complete a neuromuscular medicine fellowship.

### 2.3 Statistics and Representation

MG prevalence was reported per 100,000 Medicare FFS beneficiaries. MG prevalent cases were compared to BCNMP by state in primary analysis and by census regions and census divisions in secondary analyses. MG:BCNMP between timeframes for census regions and divisions were tested using Poisson log‐linear regression. Significant values of Wald chi‐square tests with a *p* < 0.001 were reported, adjusting for multiple comparisons. Ratios of MG Medicare FFS patients per BCNMP (MG:BCNMP) were analyzed as quintiles for group classification and geographic visualization. Maps were generated using MapChart [9].

A secondary calculation of BCNMP per population was determined using publicly available census data for these timeframes [10].

### 2.4 Standard Protocol Approvals, Registrations, and Patient Consents

This study was approved by the Institutional Review Board (IRB) at the Cleveland Clinic (IRB project number 21–1025) with data use agreements with participating institutions.

## 3 Results

The number of MG cases identified between 2012 and 2013 was 29,038 and 35,691 between 2018 and 2019; MG prevalence increased in all states, regions, and divisions (Table 1). Regional MG prevalence among the Medicare FFS population was similar in the Northeast, Midwest, and South regions, increasing from an average of 103.4 to 125.6 per 100,000 from 2012 to 2013 to 2018 to 2019. Prevalence was lowest in the West region at both timeframes.

**TABLE 1:** MG prevalence and BCNMP number by census region and division.

	MG prevalence (per 100,000 Medicare FFS population)		BCNMP		MG Medicare FFS patients per BCNMP	
	2012–2013	2018–2019	2012–2013	2018–2019	2012–2013	2018–2019
Regions						
Northeast^1^	105.4	125.6	158	226	36.0	29.8*
Midwest^2^	103.3	123.6	147	188	48.2	44.1
South^3^	104.4	126.8	178	235	67.9	63.7
West^4^	77.5	92.7	102	157	40.5	35.8
Divisions						
New England1^1^	109.2	127.0	51	82	35.2	25.3
Mid Atlantic^1^	103.7	125.0	107	144	36.4	32.4
E N Central^2^	106.2	128.0	82	103	61.3	56.6
W N Central^2^	97.0	114.3	65	85	31.6	29.0
S Atlantic^3^	107.6	129.6	98	130	69.9	66.0
E S Central^3^	100.3	128.8	29	32	69.9	78.7*
W S Central^3^	100.7	119.7	51	73	63.0	53.2
Mountain^4^	81.9	100.4	36	57	40.9	37.9
Pacific^4^	75.3	88.5	66	100	40.2	34.6

*Note:* Superscript numbers indicate which divisions comprise which regions. *Significant (*p* < 0.001) change over time.

Abbreviations: BCNMP: board certified neuromuscular physicians; E N Central, East North Central; E S Central, East South Central; FFS: fee for service; S Atlantic, South Atlantic; W N Central, West North Central; W S Central, West South Central.

BCNMP across the US increased from 585 in 2012–2013 to 806 in 2018–2019. BCNMP increased in all regions and divisions (Table 1). Six states had no BCNMP at both timeframes: AK, DE, ID, SD, RI, and WY in 2012–2013 and AK, DE, ID, ME, SD, and WY in 2018–2019.

**FIGURE 1:**
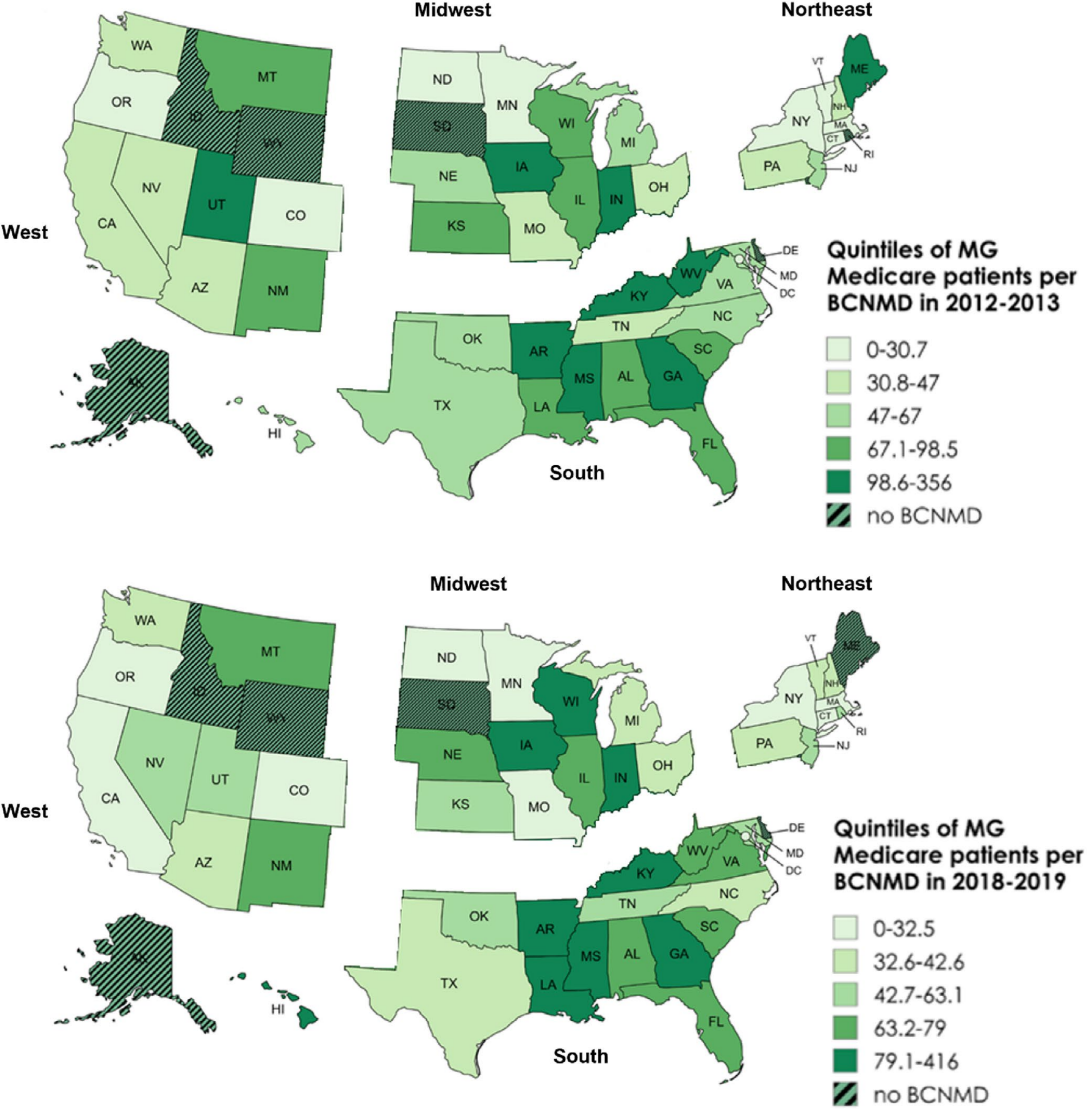
Geographic variability in ratio of MG Medicare FFS patients per BCNMP. Distribution of MG Medicare patients per BCNMP by quintile in 2012–2013 (top). There are 28,980 MG Medicare FFS patients and 586 BCNMP represented. Distribution of MG Medicare FFS patients per BCNMP by quintile in 2018–2019 (bottom). There are 35,635 MG Medicare FFS patients and 806 BCNMP represented. The darker the color, the higher the ratio of MG Medicare FFS patients per physician.

Overall MG:BCNMP across the country decreased from 49.5 to 44.3. Figure 1 demonstrates the geographic variability in ratio by state. Table 1 lists the geographic breakdown by census region and census division. The Northeast region had the largest reduction in MG:BCNMP from 36.0 in 2012–2013 to 29.8 in 2018–2019. The South region had the largest ratio at both timeframes. East South Central was the only division that demonstrated an increase in MG:BCNMP across timeframes.

**FIGURE 2:**
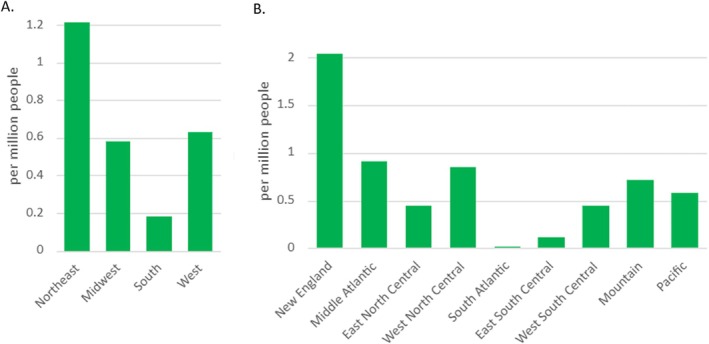
Growth of BCNMP per population 2013 to 2019. Change in number of BCNMP per million people by region (A) and division (B) between 2013 and 2019.

Figure 2 demonstrates BCNMP growth per population in regions and divisions. BCNMP per population increased most in the Northeast region and least in the South Region. Two of the three southern divisions saw the lowest increases of BCNMP.

## 4 Discussion

This study demonstrates geographic variability in the number of BCNMP compared to the prevalence of Medicare MG patients. Similar geographic variability is reported between all neurologists and neurologic patients [2]. During the period of observation, MG prevalence increased uniformly in all geographic regions; the absolute number of MG cases was lowest in the West region reflecting the lower median age in this region [7]. Medicare data may underestimate prevalence by at least 35%; 30%–65% of MG patients are age 65 years or older [5, 11]. Similar to other studies, the lowest ratios of BCNMP:MG patients were observed in the South region and the highest ratios in the Northeast region. The percentage of neurologists among physicians has been stable as well as the percentage of graduates pursuing neurology or PMR residency [12, 13]. Limited data collected since implementation of a neuromuscular fellowship match suggests 8%–13% of pooled graduating neurology and PMR residents pursue a neuromuscular medicine fellowship [14, 15, 16, 17, 18]. The ideal ratio of BCNMP to MG patients is unknown. Further work is needed to understand the ideal physician workforce size to care for the growing number of MG patients.

The MG treatment landscape is rapidly changing; seven new immunotherapies spanning multiple therapeutic classes have been approved by the U.S. Food and Drug Administration since 2018 [19]. As treatment regimens increase in complexity and cost, mismatch between neuromuscular experts and MG patients may lead to disparities in care. To utilize new treatments, physicians must be facile with new mechanisms of action and side effect profiles, and have access to the infrastructure to administer and monitor patients treated with new agents. Early evidence suggests that treatment of MG patients by a neuromuscular expert results in fewer unwanted outcomes [20]. These considerations will also be relevant to other rare neuromuscular disorders, such as muscular dystrophies, as new genetic treatments strategies become available.

Geographic differences in MG:BCNMP may preferentially impact certain populations. The south has the highest black population in the country. Health disparities are described among southerners (HIV and obesity rates) and black Americans (prevalence of heart disease and cancer, mortality secondary to vascular disease); disparities are exaggerated among elderly minorities, a population growing in MG prevalence [3, 4, 21, 22]. Recent data demonstrate that race and sex may be associated with less access to acute MG care and some treatments [23, 24, 25]. Another study showed disparities in MG clinical trial enrollment in the same geographic regions in which we observed the lowest numbers of BCNMP [26]. While a full discussion of ways to reduce healthcare inequities in the US is beyond the scope of this study, reducing geographic variability in neuromuscular specialist location may be helpful in this effort.

Though multiple states remained without BCNMP at both timeframes, at the time of submission only two states (ME and WY) remain without BCNMP. The states with the highest MG:BCNMP ratios include those largest by area, which may suggest an additional barrier of proximity. Telehealth is growing in use; the MG Core Exam was developed to facilitate telemedicine for MG patients [27]. While telehealth helps distribute clinicians to broader regions, it does not solve the issue of access to newer treatments in regions without trained experts and facilities who are able to administer these infusion therapies.

While our data identifies potential geographic differences between neuromuscular physicians and MG patients, the analysis likely underestimates the number of physicians qualified to care for MG patients. General neurologists with large cohorts of MG patients, ABPN CNL certified physicians, and ABEM certified physicians who may be well qualified to care for MG patients were excluded from analysis; it is not possible to distinguish MG and NM experts among these groups from those that are not, for example, CNL epilepsy experts, with ABPN VerifyCert. Including these groups posed a risk to overestimating the number of NM experts. We did include BCNMP through the ABPMR pathway as they could be identified with Certified Physician Search.

This study suggests that BCNMP density is variable across the United States despite uniformly increasing MG prevalence and an evolving therapeutic landscape. We hope these data inspire strategies to equalize the density of neuromuscular specialists and to provide a basis for future studies to assess how geographic variations in BCNMP density influence healthcare access and patient outcomes.

## Author Contributions

**Lauren Herrera:** writing – original draft, writing – review and editing, methodology, visualization, data curation. **Yuebing Li:** writing – review and editing, conceptualization, investigation, funding acquisition, supervision. **Jesse D. Schold:** writing – review and editing, conceptualization, methodology, formal analysis. **David Bruckman:** writing – review and editing, writing – original draft, formal analysis, data curation, methodology. **Michael K. Hehir:** writing – review and editing, investigation, conceptualization, supervision, methodology. **Ikjae Lee:** writing – review and editing, conceptualization, methodology, formal analysis. **Benjamin Claytor:** writing – review and editing, formal analysis. **Nicholas Silvestri:** writing – review and editing, formal analysis.

## Funding

This study received research grant support from Argenx BVBA. We would like to thank Mr. Ted Geiger for his generous support, which provided part of the funding to conduct this project.

## Disclosure

L. Herrera and D. Bruckman report no disclosures relevant to the manuscript. I. Lee has received research funding from the National Institute of Health and has received consultant fees from Regeneron and Argenx pharmaceuticals. J. Schold and B. Claytor report no disclosures relevant to the manuscript. N. Silvestri has consulted for Amgen, Alexion, Annexon, Argenx, Immunovant, Johnson & Johnson, and UCB. Y. Li has consulted for Amgen, Argenx, and Vertex. M. Hehir has consulted for Alexion, Argenx, UCB Pharma, and Janssen for unrelated myasthenia gravis (MG) projects and has a grant from the University of Vermont Medical Center for unrelated research in MG.

## Ethics Statement

We confirm that we have read the Journal's position on issues involved in ethical publication and affirm that this report is consistent with those guidelines.

## Conflicts of Interest

The authors declare no conflicts of interest.

## Data Availability

The data that support the findings of this study are deidentified and available from the Centers for Medicare and Medicaid Services (CMS) and Research Data Assistance Center (ResDAC). Restrictions apply to the availability of these data, which were used under license for this study. Data are available from the author(s) with the permission of CMS and ResDAC.
